# Functional Analysis of *DNMT1* SNPs (*rs2228611* and *rs2114724*) Associated with Schizophrenia

**DOI:** 10.1155/2021/6698979

**Published:** 2021-03-31

**Authors:** Sonal Saxena, Pranay Amruth Maroju, Sumana Choudhury, Vidhya Chitta Voina, Poonam Naik, Kavitha Gowdhaman, Poornima Kkani, Kiranmai Chennoju, S. Ganesh Kumar, C. Ramasubramanian, G. Prasad Rao, Trinath Jamma, Kumar Pranav Narayan, K. Naga Mohan

**Affiliations:** ^1^Department of Biological Sciences, Birla Institute of Technology and Science, Pilani – Hyderabad Campus, Hyderabad, India; ^2^Department of Zoology, Thiagarajar College, Madurai, India; ^3^Department of Psychiatry, M.S. Chellamuthu Trust and Research Foundation, Madurai, India; ^4^Asha Hospital Institute of Medical Psychology, Hyderabad 500034, India; ^5^Centre for Human Disease Research, Birla Institute of Technology and Science, Pilani – Hyderabad Campus, Hyderabad, India

## Abstract

A recent study showed the association of minor alleles of *rs2228611* (T allele) and *rs2114724* (T allele) of *DNMT1* with schizophrenia (SZ) and suggested their effects on splicing of the transcripts. We performed a replication study using 310 controls and 304 SZ patients and confirmed the association of the homozygous minor allele genotypes with SZ (*P* = 0.04 for *rs2114724* and *P* = 0.007 for rs*2228611*). This significant association persisted after Bonferroni correction when the previously published data of 301 controls and 325 patients were also considered (*P* ≤ 0.0002). In addition, we found that the proportion of male patients with homozygous minor alleles at *rs2114724* was significantly higher than that of females (*P* = 0.002). When haplotype analysis of both loci was performed, we observed a significant association of *T/T*–*T/T* and *T/T*–*C/T* (*P* = 0.04) haplotypes with SZ. To gain insights into the functional effects of the two SNPs on the levels of *DNMT1* transcripts, quantitative real-time PCR experiments were performed using peripheral blood monocytes from 10 individuals each with *T/T*–*T/T* (homozygous minor allele), *C/T*–*C/T* (heterozygous), and *C/C*–*C/C* (homozygous major allele) haplotypes. Independently, the levels of DNMT1 protein were also compared in three individuals each by immunofluorescence. These results suggest that neither *DNMT1* transcript nor the protein levels were significantly different in the peripheral blood monocytes among the individuals studied for the three groups. Taken together, our results confirm that the two minor alleles in homozygosity are associated with SZ but with no discernible effects on transcript or protein levels of *DNMT1* in the peripheral blood monocytes of the small number of samples tested.

## 1. Introduction

Schizophrenia (SZ) is a complex disorder with a worldwide incidence of ∼0.7% and multiple etiologies that include genetic, environmental, and epigenetic mechanisms [1–4]. A number of family and case-control studies identified many single-nucleotide polymorphisms (SNPs) and copy-number variants (submicroscopic deletions and duplications) as significantly associated with SZ [5, 6]. Evidence for the involvement of epigenetic mechanisms in SZ comes from twin studies wherein monozygotic (MZ) twins show 41–65% concordance [1]. Of the different epigenetic processes in mammals, DNA methylation which involves addition of methyl group at the 5^th^ carbon of cytosine is the most well studied [7]. DNA methylation is established by *de* novo methyltransferases DNMT3A and DNMT3B and maintained by DNMT1. DNMT3L has no catalytic activity but regulates *de novo* methylation [8].

**Figure 1:**
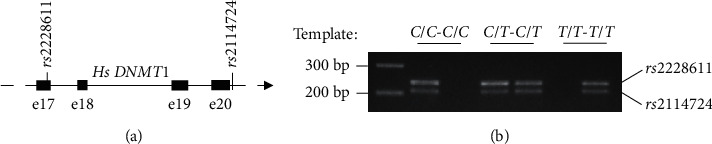
ARMS-PCR-based detection of minor and major alleles in controls and schizophrenia patients. (a) Location of the two loci in exon 17 (e17) and intron 20, respectively. (b) Validation of ARMS-PCR in distinctive identification of major and minor alleles at both loci.

Recently, SNPs of *DNMTs* were studied among SZ patients and controls from South India [9]. These results showed a significant association of minor alleles of *DNMT1* (*rs2114724*, *P* = 0.02, and *rs2228611,P* = 0.002), *DNMT3B* (*rs2424932* in SZ males, *P* *=* 0.006; *rs1569686* in early onset as well as family history, *P* = 0.03), and *DNMT3L* (*rs2070565* in an early onset of SZ as well as family history, *P* = 0.003). Among these, the minor alleles of *rs2114724* and *rs2228611* (Figure 1(a)), which are the focus of the current study, have been suggested to affect splicing of the *DNMT1* transcript. Here, we performed a replication study to validate the association of the two *DNMT1* minor alleles using an independent collection of 310 controls and 304 patients. Because defectively spliced or unspliced mRNAs in eukaryotic cells are subjected to degradation and an overall reduction of the transcript levels [10], we compared the levels of *DNMT1* transcripts and proteins among individuals with three haplotypes (homozygous for both minor alleles, homozygous for both major alleles, and heterozygous). The results are discussed here.

## 2. Materials and Methods

### 2.1. Patient and Control Samples

This work was approved by the Institutional Human Ethics Committees of the participating institutions. Normal controls and SZ patients were identified by qualified psychiatrists using DSMV criteria [11]. The controls were age- and sex-matched and did not have any neuropsychiatric conditions. After obtaining informed consent, 5–10 ml blood was drawn by venipuncture and the peripheral blood monocytes were isolated by using Histopaque 1077 (Merck, USA) solution. The purified monocytes were washed with 1X phosphate-buffered saline (1X PBS: 37 mM NaCl, 2.7 mM KCl, 10 mM Na_2_HPO_4_, and 1.8 mM KH_2_PO_4_), used for isolation of genomic DNA using SDS-proteinase K method [12] and total RNA using RNeasy Mini kit (Qiagen, Germany). Total RNAs were reverse-transcribed using SuperScript IV cDNA synthesis kit (Thermo Fisher, USA), and the resultant cDNAs were amplified using primers for *ACTIN* and *DNMT1* by real-time PCR (see below).

### 2.2. SNP Genotyping and Analysis

Genomic DNAs from 20 normal individuals were used to amplify regions containing the *rs2114724* and *rs2228611* loci. Primer sequences and annealing temperatures are given in Supplementary Table 1. For amplification, 10 ng each of the genomic DNAs was used with 5 *µ*M each of the primers in a volume of 10 *µ*l. Amplification conditions include one cycle of denaturation at 95°C for 3 min, followed by 40 cycles of PCR (95^0^C: 30 sec; 55°C: 30 sec; 68°: 30 sec) and one cycle of final extension at 68°C for 3 min. The PCR products were gel-purified using Qiaquick Gel-purification kit (Qiagen) and sequenced by Sanger's dideoxy sequencing method. Using the sequencing data, individuals with homozygous major allele, homozygous minor allele, and heterozygous genotypes at both loci were identified. The corresponding genomic DNAs were used to develop Amplification Refractory Mutation System (ARMS) by designing primers (Supplementary Table 1) that can distinguish the major and minor alleles by PCR [13]. Amplification conditions include one cycle of denaturation at 95°C for 3 min, followed by 40 cycles of PCR (95°C: 30 sec; 55°C: 15 sec; 68°C: 20 sec) and one cycle of final extension at 68°C for 3 min.

Validation of the ARMS-PCR primers was performed using the same genomic DNA samples that were sequenced. Following validation, genomic DNAs from 310 controls and 304 patients were amplified by ARMS-PCR, and the observed amplification patterns were used to deduce the genotype and allele frequencies. The number of controls and patients with a specified genotype or haplotype was used to perform a Fisher's *t-*test (https://www.graphpad.com/quickcalcs/contingency1/) using the two-tailed method and calculate odds ratios (https://www.medcalc.org/calc/odds_ratio.php) [14]. *P* values of ≤0.05 were taken as significant. For a given genotype that is significantly associated with SZ, penetrance estimates were made using CalPen, an online tool [15].

### 2.3. Real-Time PCR Analysis

cDNAs were prepared from PBMCs of 10 normal individuals each with the three haplotypes (homozygous for major alleles at both loci, heterozygous for both loci, and homozygous for minor alleles at both loci) and amplified with primers for *DNMT1* and *β*-*ACTIN* transcripts (Supplementary Table 1) using iTaq Universal SYBR Green Supermix (Bio-Rad, USA) and QuantStudio3 Real-time PCR System (Thermo Fisher, USA). *DNMT1* expression was normalized with *β*-*ACTIN* as an endogenous control to obtain ΔCt values. The average of ΔCt values from ten individuals with homozygous major haplotype was used as a reference to calculate ΔΔCt for all the 30 samples with the three haplotypes. Relative abundances of transcripts were then determined by calculating 2^−ΔΔCt^ values [16]. One-way analysis of variance (ANOVA) was performed using Microsoft Excel to identify any significant differences in *DNMT1* transcript levels among the three groups [17].

### 2.4. Immunocytochemistry

Purified PBMCs were washed and resuspended in 1X PBS and diluted to ∼10^6^ cells/ml. One millilitre of each of the cell suspensions was transferred into 6-well dishes containing coverslips and left stationary for 30 min at room temperature to allow attachment of cells [18]. The nonadherent cells were removed by aspiration, and the attached cells were fixed for 10 minutes with 500 *μ*l of 4% paraformaldehyde (Merck, USA) added gently along the side of the well. The fixed cells were washed with 1X PBS, permeabilized with 500 *μ*l of 0.5% Triton X-100 (HiMedia, India) for 10 min, washed with 1X PBS for 5 min, and blocked with 500 *μ*l of 1% BSA (HiMedia, India) for 30 minutes. Primary antibodies for DNMT1 (Thermo Fisher, 60B1220.1) and HISTONE H3 (Cell Signaling Technologies, 4499) diluted in 1% BSA were added to the dishes and were incubated overnight at 4°C. The cells were washed again with 1X PBS and incubated with fluorescently labelled secondary antibodies (Cell Signaling Technologies, 4409 and 4412) for one hour at room temperature. Washings were repeated twice with 1X PBS, counterstained with DAPI (Thermo Fisher, D1306) for 5 min, washed thrice with 1X PBS, and mounted onto glass slides for visualization using a confocal microscope (Leica: TCSSP8). The fluorescence intensities were quantified using the Leica Las-X software, using HISTONE H3 signals as reference. After normalization of data for signals obtained with HISTONE H3, the relative signals for DNMT1 in 20 random cells for each individual were used for comparisons. The DNMT1 protein levels among individuals with the three haplotypes were compared by Student's *t*-test (two-tailed), and *P* ≤ 0.05 was taken as statistically significant.

## 3. Results and Discussion

We established an ARMS-PCR to achieve allele-specific amplification so that samples containing only minor alleles (homozygous minor alleles), only major alleles (homozygous major alleles), and both the alleles (heterozygous) at the two loci were identified reliably (Figure 1(b)). We employed ARMS-PCRs for genotyping 310 controls and 304 patients (Materials and Methods).

The genotypes of all 614 individuals were classified as homozygous major allele or homozygous minor allele or heterozygous, and the number of individuals for each genotype was tabulated (Table 1). From these genotypes, the minor allele frequencies were estimated for *rs2114724* as 0.43 for controls and 0.45 for patients. The number of controls with the minor allele was not significantly different from patients. On the other hand, the number of patients with the minor allele for *rs2228611* (frequency = 0.47) was significantly higher (*P* = 0.04) than controls (frequency = 0.41). Fisher's *t-*tests were performed to identify whether any genotype frequency is significantly different between controls and patients. For example, 158 out of 310 controls have *C*/*T* genotype for *rs2114724* with the remaining controls (152) having either *C*/*C* or *T*/*T* genotypes. In the case of patients, 125 out of 304 patients have *C*/*T* genotype and the remaining 179 have either *C*/*C* or *T*/*T* genotype. Using this information, when a two-tailed Fisher's *t-*test was performed, a *P* value of 0.01 was obtained, indicating that the proportion of *C*/*T* individuals among controls (51%) was significantly higher than that in patients (41%). The odds ratio for this genotype was 0.7, meaning that the odds for *C*/*T* genotype to be a control is higher than being schizophrenic. Similar analysis suggested that homozygous minor allele genotypes for *rs2114724* (*P* *=* 0.04; odds ratio = 1.5253) and *rs2228611* (*P* *=* 0.007; odds ratio = 1.7) were significantly higher in patients than in controls. A combined analysis with the previously published data [9] amounting to a total of 611 controls and 629 patients revealed a more significant association of the minor alleles of both loci with SZ after Bonferroni correction (*P* ≤ 0.0002) with individual odds ratios of 1.73 (*rs2114724*) and 1.83 (*rs2228611*), respectively.

We next examined whether there was any gender-specific difference in the distribution of the minor allele-associated genotypes in patients (Table 2). For *rs2114724,* the proportion of minor allele homozygous males (52 out of 170) was significantly higher (*P* = 0.002) than that in females (22 out of 134). There was no such gender-specific difference in the case of homozygous minor allele genotypes for *rs228611*.

Haplotype analysis between controls and patients revealed a significant association of homozygous minor allele genotype of *rs2228611* (*T*/*T*) with either homozygous minor allele genotype (*P* *=* 0.04; odds ratio = 1.52) or heterozygous genotype of *rs2114724* (*P* = 0.04; odds ratio = 4.7) in patients (Table 3).

**Figure 2:**
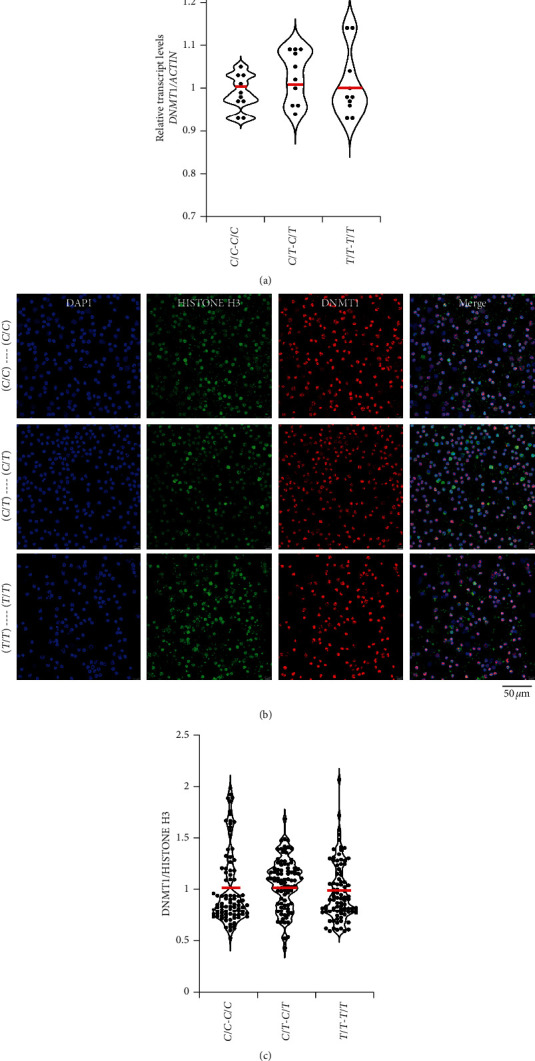
Evaluation of *DNMT1*. (a) Violin plots showing relative transcript levels in peripheral blood monocytes of individuals with the haplotypes indicated on the *X*-axis. (b) Immunofluorescence of peripheral blood monocytes with antibodies to HISTONE H3 (green) and DNMT1 proteins (red). (c) Quantification of the signals obtained for DNMT1 after normalization for the signals obtained with HISTONE H3 in peripheral blood monocytes of three individuals each for the genotypes indicated.

Minor alleles of both *rs2228611* and *rs2114724* have been suggested to affect the splicing process of the *DNMT1* mRNA [9]. Since mRNAs that are aberrantly spliced or fail to undergo successful splicing are unstable and degraded [10], we measured *DNMT1* mRNA levels in peripheral blood monocytes of 10 individuals each with *C*/*C*–*C*/*C*, *C*/*T*–*C*/*T*, and *T*/*T*–*T*/*T* haplotypes. Although the quantitative real-time PCR data revealed variation in the transcript levels within a haplotype, there was no difference in the average *DNMT1* transcript levels between the three haplotypes (Figure 2(a)).

As an independent approach, the DNMT1 protein levels were compared among three individuals each with the three haplotypes by immunocytochemistry (Figure 2(b)). As in the case of *DNMT1* transcripts, the distribution of the relative DNMT1 protein amounts varied within a haplotype and there was no difference in the protein levels among the three haplotypes (Figure 2(c)). These results taken together with the transcript levels compared by real-time PCR indicate that the minor allele haplotype (*T*/*T*–*T*/*T*) may not be associated with any identifiable decrease in the *DNMT1* transcript or protein levels.

## 4. Conclusions

Minor alleles of *rs2114724* and *rs2228611* have been reported to be associated with SZ, gastric, ovarian, and breast cancers, hypertension, and hearing loss [19–24]. The results reported here support a significant association of these alleles with SZ. Our observation is based on a total of 611 controls and 629 patients, and given the small size of samples studied, more studies are needed to confirm these findings. Contrary to the prediction that the minor alleles affect the splicing process, there was no discernible change in the transcript or protein levels in the PBMCs of individuals with only minor alleles at both loci. Since the sample size tested here is small, future studies involving a larger set of samples may identify events where DNMT1 transcript or protein levels are affected. It is also possible that the effects of these minor alleles are specific to neurons. In this context, a similar functional analysis on postmortem brain samples is warranted. The effects of these minor alleles may still end up to be too small to be detected experimentally even in the brain. Whether such small effects, if at all present, along with any other variants elsewhere in the *DNMT1* gene or other genes result in an identifiable molecular abnormality related to SZ requires further investigation.

## Figures and Tables

**Table 1:** Analysis of *rs2114724* and *rs2228611* polymorphisms in controls and schizophrenia patients.

SNP	Genotype	Present study				Saradalekshmi et al. [9]		Combined				
		Controls	Patients	Odds ratio (CI)	*P* value	Controls	Patients	Controls	Patients	Odds ratio (CI)	Bonferroni-corrected *P* value	Penetrance (CI)
*rs2114724*	*C*/*C*	98 (0.32)	105 (0.35)	1.14 (0.81–1.6)	0.44	98 (0.33)	97 (0.30)	196 (0.32)	202 (0.32)	1.00 (0.79–1.27)	1.000	—
	*C* / *T*	158 (0.51)	125 (0.41)	0.67 (0.49–0.93)	0.01	160 (0.53)	147 (0.45)	318 (0.52)	272 (0.43)	0.70 (0.56–0.88)	0.004	—
	***T*/*T***	**54 (0.17)**	**74 (0.24)**	**1.52 (1.03**–**2.26)**	**0.04**	**43 (0.14)**	**81 (0.25)**	**97 (0.16)**	**155 (0.25)**	**1.73 (1.31**–**2.29)**	**0.0002**	**0.011 (0.008**–**0.015)**

*rs2228611*	*C*/*C*	107 (0.35)	100 (0.33)	0.93 (0.66–1.3)	0.73	113 (0.38)	97 (0.30)	220 (0.36)	197 (0.32)	0.81 (0.64–1.03)	0.164	—
	*C*/*T*	147 (0.47)	121 (0.39)	0.73 (0.53–1.01)	0.07	152 (0.51)	155 (0.49)	299 (0.49)	276 (0.44)	0.82 (0.65–1.02)	0.14	—
	***T*/*T***	**56 (0.18)**	**83 (0.28)**	**1.7 (1.16 to 2.50)**	**0.007**	**34 (0.11)**	**68 (0.21)**	**90 (0.15)**	**151 (0.24)**	**1.83 (1.37**–**2.44)**	**<0.0002**	**0.011 (0.008–0.016)**

The major and minor alleles for *rs2114724* and *rs2228611* are *C* and *T*, respectively. CI: confidence interval. Underlined *P* values indicate that the genotypes occur at high frequencies in controls than in patients. *P* values in bold indicate that the genotypes occur at high frequencies in patients than in controls. The total number of controls in the present and previous studies was 310 and 301, respectively, whereas the number of patients was 304 and 325, respectively.

**Table 2:** Proportion of *T/T* homozygotes for *rs2114724* among male and female patients.

Genotype	Males	Females	Chi-square analysis
*T*/*T*	52 (41) [2.95]	22 (33) [3.67]	Total *χ*^2^ = 12.22; degrees of freedom = 2; *P* value = 0.002
*C/T*	71 (70) [0.01]	54 (55) [0.02]	
*C/C*	47 (59) [2.44]	58 (46) [3.13]	

Individual *χ*^2^ values obtained using a 2 × 3 contingency are shown in square brackets. The expected number of males and females is given in parentheses.

**Table 3:** Haplotype analysis of alleles at*****rs2114724* and *rs2228611* in controls and schizophrenia patients.

Haplotype (*rs2114724*–*rs2228611*)	Controls	Patients	Odds ratio (CI)	*P* value	Penetrance (CI)
(*C*/*C*) ---- (*C*/*C*)	96 (0.31)	97 (0.32)	1.04 (0.74–1.46)	0.86	—
(*C*/*C*) ---- (*C*/*T*)	2 (0.01)	8 (0.03)	4.16 (0.88–19.76)	0.06	—
(*C*/*C*) ---- (*T*/*T*)	0 (0.00)	0 (0.00)	—	—	—
(*C*/*T*) ---- (*C*/*C*)	11 (0.04)	3 (0.01)	0.27 (0.08–0.98)	0.06	—
( *C* / *T* ) ---- ( *C* / *T* )	145 (0.47)	113 (0.37)	0.67 (0.49–0.93)	0.01	—
(*C*/*T*) ---- (*T*/*T*)	**2 (0.01)**	**9 (0.03)**	**4.70 (1.01**–**21.92)**	**0.04**	**0.025 (0.005**–**0.182)**
(*T*/*T*) ---- (*C*/*C*)	0 (0.00)	0 (0.00)	—	—	—
(*T*/*T*) ---- (*C*/*T*)	0 (0.00)	0 (0.00)	—	—	—
(*T*/*T*) ---- (*T*/*T*)	**54 (0.17)**	**74 (0.24)**	**1.52 (1.03**–**2.26)**	**0.04**	**0.01 (0.006**–**0.016)**

Underlined *P* values indicate that the frequency of the haplotype is significantly higher in controls than in patients. *P* values in bold indicate that the haplotypes are significantly higher in patients than in controls. CI: confidence interval.

## Data Availability

The data present in the manuscript does not include any files that need to be deposited in the public repositories. All necessary information on the data collected is given in the manuscript itself.

## Supplementary Materials

Supplementary Materials Supplementary Table 1: oligonucleotides used for sequencing, ARMS-PCRs, and real-time PCR analysis .
