# Supplementary material for: Functional Analysis of DNMT1 SNPs (rs2228611 and rs2114724) Associated with Schizophrenia
Source: Genet Res (Camb). 2021 Mar 31;2021:6698979. doi: 10.1155/2021/6698979 (PMC8032507; doi:10.1155/2021/6698979)
Supplement: Supplementary Materials — Supplementary Table 1: oligonucleotides used for sequencing, ARMS-PCRs, and real-time PCR analysis . [file 6698979.f1.docx]

**Supplementary Table 1. Oligonucleotides used for sequencing, ARMS-PCRs and Real-time PCR analysis.**

| Name | Forward | Reverse | Product size (bp) |
| --- | --- | --- | --- |
| *rs2228611*_Seq | GGTTCCAGCATCTCAGAGGA | GCCTGATCTGAAGTCTGCAC | 226 |
| *rs2114724*_Seq | GGTTCCAGCATCTCAGAGGA | TGGAGAGTTATGACGAGGCC | 199 |
| *rs228611* Major | GTCTCCAGTCTTCACTCTGGTCCAC | CTCTTTCAAGACCACGGTTCCTCC | 236 |
| *rs228611* Minor | GTCTCCAGTCTTCACTCTGGTCCAT |  |  |
| *rs2114724* Major | GACTATTCCTTACCTTCAAGAGTC | CCTCGTGCCTGATCTGAAGT | 212 |
| *rs2114724* Minor | GACTATTCCTTACCTTCAAGAGTT |  |  |
| *β-ACTIN* | GCTCGTCGTCGACAACGGCTC | CAAACATGATCTGGGTCATCTTCTC | 353 |
| *DNMT1* | CCCACCAGACGCGGTGGATGAG | CAGAATGTATTCGGCAAATGAGG | 355 |
